# Decoding Post‐Stroke Cognitive Impairment After Acute Basal Ganglia Infarction: The Synergistic Role of Functional Segregation and Integration in an SVM fMRI Framework

**DOI:** 10.1002/cns.70871

**Published:** 2026-04-10

**Authors:** Shijian Chen, Jian Zhang, Liya Pan, Baohui Weng, Yijie Mo, Xuemei Quan, Gengyu Cen, Xize Jia, Yayuan Liu, Zhijian Liang

**Affiliations:** ^1^ Department of Neurology The First Affiliated Hospital of Guangxi Medical University Nanning Guangxi China; ^2^ Department of Neurology The Fourth Affiliated Hospital of Guangxi Medical University Liuzhou Guangxi China; ^3^ Department of Neurology The Second Affiliated Hospital of Guangxi Medical University Nanning Guangxi China; ^4^ Department of Neurology The People's Hospital of Guangxi Zhuang Autonomous Region, Institute of Brain and Mental Diseases, Guangxi Academy of Medical Sciences Nanning Guangxi China; ^5^ School of Psychology Zhejiang Normal University Jinhua China; ^6^ Intelligent Laboratory of Zhejiang Province in Mental Health and Crisis Intervention for Children and Adolescents Zhejiang Normal University Jinhua China; ^7^ Department of Neurology The Affiliated Minzu Hospital of Guangxi Medical University Nanning Guangxi China

**Keywords:** dynamic functional connectivity, functional magnetic resonance imaging, Ischaemic stroke, stroke, support vector machine

## Abstract

**Objective:**

To investigate whether dynamic changes in resting‐state functional MRI (rs‐fMRI) metrics can serve as sensitive biomarkers for distinguishing acute basal ganglia cerebral infarction (BGCI) patients with post‐stroke cognitive impairment (PSCI) from those without (non‐PSCI).

**Materials and Methods:**

Data on various rs‐fMRI metrics dynamic functional connectivity (dFC), dynamic amplitude of low‐frequency fluctuation (dALFF), and percent amplitude of fluctuation (PerAF) were acquired using a Siemens Prisma 3.0T scanner from 38 PSCI and 36 non‐PSCI patients, with follow‐up assessments. Functional segregation and integration were analyzed using PerAF, dALFF, and dFC. Feature extraction and selection were performed using support vector machine (SVM), followed by classifier construction and evaluation.

**Results:**

Patients with PSCI showed decreased PerAF in the left cerebellar Crus I (lCbeCru1) and increased dALFF in the right cerebellar Crus I and left lingual gyrus compared to non‐PSCI patients. Altered dFC was observed between cerebellar cognitive‐related seed regions and widespread cortical areas, with increased dFC in the right cerebellar Crus II and left cuneus, and decreased dFC primarily in the inferior frontal gyrus and superior temporal gyrus. Among single‐feature models, dFC achieved the best classification performance (AUC = 0.98, accuracy = 94.52%, sensitivity = 97.14%, specificity = 92.11%, precision = 91.89%). A combined feature model yielded the highest precision (94.12%).

**Conclusion:**

SVM‐based integration of PerAF, dALFF, and dFC features holds promise as a neuroimaging biomarker for PSCI in patients with BGCI. This approach may support more precise early rehabilitation strategies in clinical practice.

## Graphical abstract

Post‐stroke cognitive impairment (PSCI) currently lacks reliable neuroimaging biomarkers for diagnosis. PSCI patients exhibit altered cerebellar‐cortical dynamics in PerAF, dALFF, and dFC, and an SVM classifier based on dFC features achieves 94.52% accuracy and 0.98 AUC, outperforming single‐metric models. This non‐invasive fMRI approach provides a promising tool for early and objective PSCI detection, facilitating timely cognitive rehabilitation and personalized intervention for stroke survivors.

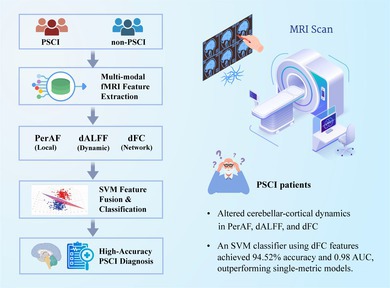

**Abbreviations**
AAL: Automated Anatomical Labeling
AUC: Area under the Curve
BGCI: Basal ganglia cerebral infarction
BOLD: Blood oxygenation level‐dependent
dALFF: Dynamic amplitude of low‐frequency fluctuation
dFC: Dynamic functional connectivity
DMN: Default mode network
FD: Frame‐wise displacement
GRF: Gaussian random field
MRI: Magnetic resonance imaging
PerAF: Percent amplitude of fluctuation
PSCI: Post‐stroke cognitive impairment
ROC: Receiver operating characteristic
ROI: Region of interest
rs‐fMRI: Resting‐state functional MRI
SVM: Support vector machine
TE: Echo time
TR: Repetition time

## 1 Introduction

Post‐stroke cognitive impairment refers to a spectrum of cognitive deficits occurring after stroke, ranging from mild subjective complaints to major neurocognitive disorder. PSCI is a highly prevalent and debilitating condition, affecting approximately 30% of stroke survivors and significantly compromising their quality of life and functional independence [1, 2]. Post‐stroke dementia (PSD), representing the most severe end of this spectrum, develops in about 7% of patients within 1‐year post‐stroke [3]. However, not all cognitive deficits following stroke meet the threshold for dementia. In fact, a substantial proportion of patients experience milder cognitive deficits. Core domains impacted include executive function, memory, attention, language, and visuospatial abilities, with executive dysfunction and memory impairment being the most frequent [4, 5]. Clinically, the anatomical location of cerebral infarction often fails to fully account for cognitive deficits. Tuladhar et al. proposed that focal lesions may disrupt neuronal signaling, leading to widespread functional alterations in remote brain regions or specific networks [6, 7].

rs‐fMRI offers a non‐invasive, radiation‐free tool with high spatial resolution for visualizing brain activity, based on blood oxygenation level‐dependent (BOLD) contrast [8]. First described by Ogawa et al. in the early 1990s, the BOLD signal reflects changes in blood oxygenation associated with neural activity. Increased neuronal firing elevates local oxygenated hemoglobin, reducing deoxyhemoglobin and shortening T2* relaxation time, thereby modulating the MR signal [9]. rs‐fMRI captures spontaneous low‐frequency fluctuations in the BOLD signal, enabling the study of intrinsic brain activity in various neurological and psychiatric conditions.

Previous rs‐fMRI studies have highlighted impaired functional connectivity within the default mode network (DMN) in PSCI patients [10, 11]. Growing evidence indicates that cerebellar Crus I and Crus II are functionally coupled with the DMN. Anatomical and functional neuroimaging studies have delineated distinct cerebro‐cerebellar loops, with posterior cerebellar regions—notably Crus I and II—forming reciprocal connections with prefrontal and parietal association cortices via the thalamus. These circuits are integral to higher‐order cognitive processes frequently impaired after stroke, such as executive function, working memory, and attentional control. Consequently, focal stroke lesions, even in subcortical structures like the basal ganglia, may disrupt these long‐range loops, leading to functional alterations in remote but connected cerebellar nodes, thereby contributing to cognitive deficits [12, 13].

Recent rs‐fMRI studies have begun to implicate cerebellar dysfunction in PSCI, reporting altered hippocampal‐cerebellar connectivity and disrupted cerebellar integration within large‐scale cognitive networks [14, 15]. However, much of this evidence relies on static connectivity measures and has not specifically interrogated the dynamic, time‐varying properties of cerebellar‐cortical communication following a strategic subcortical infarction. The aim of the present study was to investigate alterations in dFC between the cerebellum and cerebral cortex, and their relationship with cognitive deficits in PSCI. Furthermore, we employed advanced local brain activity metrics PerAF and dALFF to examine functional segregation, thus providing a comprehensive perspective on brain functional reorganization in PSCI, from local dynamics to global integration.

In recent years, machine learning approaches, particularly SVM, have gained prominence in neuroimaging. SVM is a multivariate pattern recognition technique well‐suited for high‐dimensional, nonlinear, and small‐sample‐size data [16, 17, 18]. It shows great potential in identifying optimal hyperplanes for clinical diagnosis and has been widely applied to MRI‐based classification in neurodegenerative and psychiatric disorders, including chronic post‐stroke aphasia [19] and depression [20]. Currently, PSCI diagnosis relies heavily on cognitive scales and clinical symptoms, underscoring the need for more objective biomarkers.

By utilizing a multi‐faceted approach, this study aims to advance beyond the previous reports. Firstly, we employed dynamic metrics (dFC, dALFF, PerAF) to capture the time‐varying properties of cerebellar‐cortical communication and local cerebellar activity, which may be more sensitive to early post‐stroke pathophysiology. Secondly, we specifically focused on a cohort of acute BGCI patients to elucidate network dysfunction stemming from this common and strategically located stroke subtype [21, 22]. Finally, we integrated these multi‐modal dynamic features within an SVM classifier framework to evaluate their combined utility as diagnostic biomarkers. Our overall aim was therefore to provide a more comprehensive, dynamic, and clinically translational assessment of cerebellar involvement in PSCI pathogenesis.

## 2 Materials and Methods

### 2.1 Participants

This prospective cohort study enrolled 79 patients with acute BGCI [21], including 41 with PSCI and 38 with non‐PSCI. All participants were recruited from the department of neurology at the First Affiliated Hospital of Guangxi Medical University between March 2022 and December 2023. Diagnosis of first‐ever BGCI was confirmed within 7 days of symptom onset via cranial CT or MRI, in accordance with the 2018 Chinese Guidelines for Diagnosis and Treatment of Acute Ischemic Stroke. Diagnosis also required the presence of corresponding neurological signs confirmed by diffusion‐weighted imaging (DWI). Cognitive function was assessed at the 6‐month follow‐up using a comprehensive battery that evaluated core cognitive domains, including global orientation, memory, and executive function. Patients were classified into the PSCI group if they demonstrated impairment in at least one of these core domains, while also scoring < 26 on the Montreal Cognitive Assessment (MoCA) [23, 24].

Inclusion criteria were: age 18–80 years; ability to complete neuropsychological assessments and MRI scanning; and no history of thrombolysis or endovascular therapy. Key exclusion criteria were: recurrent or multifocal infarcts; history of significant head trauma, epilepsy, or other major neurological/psychiatric disorders; MRI evidence of other pathologies (e.g., tumor, hemorrhage, or white matter lesions with a modified Fazekas score > 1 [25]); severe systemic diseases; pregnancy or lactation; absence of motor deficits; death during follow‐up; or poor‐quality MRI data. Healthy controls (HCs) were required to have no major systemic or neuropsychiatric illnesses. To control for potential confounders, this study employed strict enrollment criteria were applied: all patients had first‐ever, unilateral BGCI, and those with multifocal or large territorial infarcts were excluded. Both PSCI and non‐PSCI patient groups received standardized, guideline‐based secondary prevention pharmacotherapy (antiplatelet agents, statins, and antihypertensives as indicated). This approach aimed to minimize systematic between‐group differences in medication effects on functional network metrics.

### 2.2 Standard Protocol, Approvals, and Patient Consents

The study protocol was approved by the Institutional Review Board of the hospital. Written informed consent was obtained from all participants after a detailed explanation of the study procedures.

### 2.3 Neuropsychiatric Assessments

A comprehensive neuropsychological battery was administered by trained therapists to characterize the clinical profile of the cohort. Stroke severity and neurological deficits in patients with BGCI were quantified using the National Institutes of Health Stroke Scale (NIHSS) and the Barthel Index (BI), with motor impairment further detailed via the Fugl‐Meyer Assessment (FMA) [26, 27]. Cognitive function was assessed in all participants using the Beijing version of the MoCA and the Chinese version of the Mini‐Mental State Examination (MMSE). To account for potential mood confounders, depressive symptoms were evaluated using the 17‐item Hamilton Depression Rating Scale (HDRS) and the 9‐item Patient Health Questionnaire (PHQ‐9) [26, 28].

### 2.4 MRI Data Acquisition

All magnetic resonance imaging was performed on a 3.0T Siemens Prisma scanner equipped with a 64‐channel head–neck coil at the Imaging Center of The First Affiliated Hospital of Guangxi Medical University. Prior to scanning, all participants were screened for standard MRI contraindications. To minimize motion artifact, head positioning was stabilized with foam padding, and earplugs were provided to attenuate acoustic noise. Subjects were explicitly instructed to remain awake, keep their eyes closed, avoid structured thinking, and minimize head movement during the acquisition.

High‐resolution 3D T1‐weighted anatomical images were acquired using a gradient‐echo‐planar imaging (EPI) sequence with the following parameters: repetition time (TR) = 2300 ms, echo time (TE) = 2.98 ms, inversion time (TI) = 900 ms, slice thickness = 1 mm, voxel size = 1 × 1 × 1 mm^3^, matrix = 256 × 256, field of view (FOV) = 256 × 256 mm^2^, flip angle = 9°, 176 sagittal slices. rs‐fMRI data were obtained using a T2*‐weighted gradient‐echo planar imaging sequence sensitive to BOLD contrast: TR/TE = 2000/35 ms, slice thickness = 3 mm, voxel size = 2.6 × 2.6 × 3 mm^3^, matrix = 64 × 64, FOV = 240 × 240 mm^2^, flip angle = 90°, 40 interleaved axial slices, 186 volumes. Additionally, conventional clinical sequences, including T1‐weighted imaging (T1WI), DWI, T2‐weighted imaging (T2WI), and fluid‐attenuated inversion recovery (FLAIR), were collected for comprehensive clinical diagnosis.

Identical scanning parameters and protocols were applied to all participants, including patient groups and healthy controls. Immediately following each scan, all acquired images underwent rigorous visual quality inspection by two board‐certified neuroradiologists to rule out significant artifacts or structural abnormalities.

### 2.5 Preprocessing of rs‐fMRI Data

Preprocessing of rs‐fMRI data was performed in MATLAB R2018a (version R2018a, MathWorks Inc., Natick, MA, USA) using the Statistical Parametric Mapping software package (SPM12, Wellcome Trust Centre for Neuroimaging, London, UK; www.il.ion.ucl.ac.uk/spm) and Resting‐State fMRI Data Analysis Toolkit plus (RESTplus V1.27, http://restfmri.net/forum/restplus) [29]. The pipeline involved: discarding the first 10 volumes for signal equilibrium; slice timing correction; and head motion realignment (subjects with > 2.0 mm displacement or > 2.0° rotation were excluded). Images were then normalized to MNI space (3 × 3 × 3 mm^3^ voxels) and spatially smoothed with a 6 mm FWHM Gaussian kernel. Subsequent steps included linear detrending, regression of nuisance covariates (Friston‐24 parameters, white matter, and CSF signals) [30], and band‐pass filtering (0.01–0.08 Hz) to reduce low‐frequency drift and physiological noise [31]. Temporal scrubbing was omitted to maintain data continuity for dynamic analysis [32]. This comprehensive preprocessing pipeline, particularly the regression of nuisance signals (Friston‐24 parameters, white matter, and CSF), was implemented to mitigate the effects of non‐neural physiological noise and residual motion artifacts. Together with the stringent radiological exclusion criteria applied during enrollment, these steps aimed to reduce the potential confounding influence of pre‐existing structural pathologies and acute physiological variables on the derived functional metrics.

### 2.6 PerAF Analysis

PerAF was calculated to quantify the magnitude of BOLD signal fluctuations relative to the mean signal intensity of the time series. Analysis was performed on preprocessed fMRI data in the conventional frequency band (0.01–0.08 Hz) using RESTplus V1.27. To control for global effects, individual PerAF maps were first mean‐scaled (mPerAF) by dividing by the global mean PerAF value. Subsequently, these mPerAF maps were standardized by subtracting the global mean and dividing by the global standard deviation, resulting in *z*‐scored PerAF (zPerAF) maps used for all group‐level analyses.

### 2.7 Dynamic ALFF Analysis

dALFF was computed using the Temporal Dynamic Analysis (TDA) toolbox (http://restfmri.net/forum/restplus) within RESTplus. A sliding‐window approach with a Hamming window was employed. For each subject, the entire BOLD time series was partitioned into 127 overlapping windows, each comprising 50 TRs (100 s) with a step size of 1 TR (2 s). To enable comparison across windows and subjects, ALFF values within each window were converted to z‐scores by subtracting the global mean and dividing by the global standard deviation of the whole‐brain ALFF map for that specific window [33]. The dALFF metric for each voxel was then defined as the variance (or standard deviation) of these windowed *z*‐scored ALFF values across all 127 windows.

### 2.8 dFC Analysis

dFC was assessed using a sliding‐window approach within the conventional frequency band (0.01–0.08 Hz), implemented in the TDA toolbox of RESTplus V1.27. Six cerebellar regions of interest (ROIs) from the Automated Anatomical Labeling (AAL) atlas were selected as seeds: left and right cerebellar Crus I (lCbeCru1, rCbeCru1), Crus II (lCbeCru2, rCbeCru2), and cerebellar posterior lobe 9 (lCbe9, rCbe9). A sliding‐window approach was employed using a Hamming window to reduce spectral leakage and ensure smooth transitions at the edges of each window segment. The window length was set to 50 TRs (100 s) with a step size of 1 TR (2 s), resulting in 127 overlapping windows for the entire time series. For each window, Pearson's correlation coefficients between each seed and all other brain voxels were computed and Fisher's *z*‐transformed to improve normality, yielding a series of whole‐brain dFC matrices over time. The variability of connectivity was quantified for each voxel as the standard deviation of the z‐scored correlation coefficients across all windows, representing dFC flexibility. The resulting dFC maps were spatially smoothed with a 6 mm Gaussian kernel.

### 2.9 Feature Extraction and Selection

Following preprocessing, voxel‐wise parameter maps of PerAF, dALFF, and dFC were obtained for each participant. To identify brain regions exhibiting significant differences between PSCI and non‐PSCI groups, two‐sample *t*‐tests were conducted with Gaussian random field (GRF) correction for multiple comparisons (voxel‐level *p* < 0.005, cluster‐level *p* < 0.05, two‐tailed). Statistically significant clusters were saved as binary masks, from which the mean PerAF, dALFF, and dFC values were extracted from each significant cluster for every subject. To remove scale differences across features, these extracted values were then standardized (converted to *z*‐scores) across all subjects. The resulting standardized feature vectors served as input for the SVM classification.

The SVM classifier was implemented using the LIBSVM (https://www.csie.ntu.edu.tw/~cjlin/libsvm/) package within the MATLAB environment. To mitigate the risk of overfitting and enhance model generalizability, a linear kernel SVM was employed, allowing direct interpretation of feature weights and supporting robust classification between PSCI and non‐PSCI cohorts [34].

### 2.10 Classifier Construction and Evaluation

The extracted features were used to construct an SVM classifier for distinguishing PSCI from NPSCI patients. This was implemented using the LIBSVM toolbox in MATLAB. A linear kernel was employed to mitigate the risk of overfitting. The classifier was optimized via a grid search for the parameter *γ* (range: 2^−5^ to 2^5^), with the cost parameter (C) set to 1. Model performance was evaluated using leave‐one‐out cross‐validation (LOOCV) [35]. Classification accuracy was assessed by calculating accuracy, sensitivity, specificity, precision, and the area under the receiver operating characteristic curve (AUC) [36].

### 2.11 Statistical Analyses

Demographic and clinical characteristics were compared between groups using independent samples *t*‐tests for normally distributed continuous variables, Mann–Whitney U tests for non‐normally distributed variables, and chi‐square tests for categorical data as appropriate.

For voxel‐wise comparisons of PerAF, dALFF, and dFC maps, two‐sample *t*‐tests were conducted with age, sex, years of education, and mean framewise displacement included as covariates. Statistical maps were corrected for multiple comparisons using Gaussian random field theory, with a voxel‐level threshold of *p* < 0.001 and a cluster‐level threshold of *p* < 0.05. Peak coordinates of significant clusters were localized anatomically using the Automated Anatomical Labeling atlas in xjView (http://www.alivelearn.net/xjview).

To explore clinical relevance, parameter values from significant clusters were extracted and subjected to partial correlation analyses with key clinical measures (e.g., FMA, NIHSS, BI, MoCA, PHQ‐9, HAMD), while controlling for age, sex, and education. A significance level of *p* < 0.05 was applied for all inferential tests. All statistical analyses were performed using SPSS 26.0 (IBM, Armonk, NY, USA).

## 3 Results

### 3.1 Demographic and Clinical Features

This study prospectively and consecutively enrolled 41 patients with acute BGCI and PSCI, along with 38 BGCI patients without PSCI (non‐PSCI) as a control group. Following the strict application of exclusion criteria, three patients from the PSCI group and two from the non‐PSCI group were excluded during preprocessing due to excessive head motion (> 2 mm or 2°). Consequently, 38 PSCI patients and 36 non‐PSCI patients were included in the final analysis.

Comparisons of demographic and clinical data using independent two‐sample *t*‐tests, Mann–Whitney *U* tests, and chi‐square tests revealed statistically significant differences between the PSCI and non‐PSCI groups in age at onset, years of education, and MoCA scores (*p* < 0.05). In contrast, no significant differences were observed for gender, mean frame‐wise displacement (FD), cerebrovascular risk factors, NIHSS scores, total FMA and its upper/lower limb sub‐scores, BI index, mRS scores, PHQ‐9, or HAMD scores (*p* > 0.05). Details are presented in Table 1.

**TABLE 1:** Demographic and clinical characteristics of PSCI and NPSCI groups.

Characteristics	PSCI (*n* = 38)	NPSCI (*n* = 35)	*t*/*χ* ^2^/*z*	*p*
Gender, male	28.00 (73.60)	29.00 (82.60)	0.896	0.344^c^
Age, years	58.20 ± 8.10	53.26 ± 9.10	2.447	0.017^a,^*
Educational level, years	9 (8.25–12.00)	12.00 (9.00–15.00)	−2.833	0.005^b,^*
Mean FD	0.06 (0.04–0.11)	0.07 (0.05–0.11)	−0.994	0.320^b^
High risk factor				
Hypertension	25.00 (65.80)	23.00 (65.70)	0.001	0.995^c^
Diabetes	7.00 (18.40)	8.00 (22.90)	0.220	0.639^c^
Hyperlipdemia	11.00 (28.90)	9.00 (25.70)	0.096	0.757^c^
Smoking	17.00 (44.70)	20.00 (57.10)	1.122	0.290^c^
Drinking	16.00 (42.10)	19.00 (54.30)	1.083	0.298^c^
Clinical examinations				
NIHSS	3.00 (2.00–6.00)	3.00 (2.00–5.00)	−0.162	0.871^b^
FMA	79.00 (69.00–88.50)	86.00 (64.00–93.0)	−1.420	0.156^b^
UL‐FMA	42.25 (15.90–53.00)	58.00 (40.00–61.00)	−1.271	0.204^b^
DL‐FMA	28.00 (25.50–30.00)	30.00 (24.00–33.00)	−1.353	0.176^b^
MoCA	15.00 (12.70–18.00)	27.00 (26.00–29.00)	−7.379	0.001^b,^*
BI	80.00 (55.00–95.00)	90.00 (65.00–100.0)	−1.789	0.074^b^
mRS	2.00 (1.00–3.00)	2.00 (1.00–3.00)	−0.740	0.459^b^
PHQ‐9	5.00 (1.75–9.50)	6.00 (3.00–9.00)	−0.809	0.419^b^
HAMD	5.00 (2.00–14.00)	6.00 (3.00–12.00)	−0.416	0.677^b^

*Note:* Data are presented as mean ± standard deviation, median (interquartile range), or number of participants (%), as appropriate. Significant between‐group differences (*p* < 0.05) are indicated by an asterisk (*). Group comparisons were performed using (a) independent samples *t*‐test, (b) Mann–Whitney *U* test, or (c) Chi‐square test.

Abbreviations: BI, Barthel Index; DL‐FMA, Lower Limb FMA; FD, Framewise Displacement; FMA, Fugl‐Meyer Assessment; HAMD, Hamilton Depression Scale; MoCA, Montreal Cognitive Assessment; mRS, modified Rankin Scale; NIHSS, National Institutes of Health Stroke Scale; NPSCI, non‐post‐stroke cognitive impairment; PHQ‐9, Patient Health Questionnaire‐9; PSCI, post‐stroke cognitive impairment; UL‐FMA, Upper Limb FMA.

### 3.2 Frequency‐Specific Percent Amplitude of Fluctuation (PerAF)

A pivotal finding emerged from our whole‐band analysis: PSCI patients exhibited widespread reduction in PerAF values in the left superior cerebellar peduncle compared to the non‐PSCI group. This phenomenon was consistently observed not only in the conventional frequency band but also in the more functionally specific slow‐4 and slow‐5 sub‐bands.

Specifically, significantly decreased PerAF in the left superior cerebellar peduncle was detected in the conventional band in the PSCI group. Notably, this reduction remained stable across the distinct high‐ and low‐frequency sub‐bands—slow‐5 and slow‐4, which are respectively associated with maintenance of basal neural activity and higher‐order cognitive processing. This consistent pattern across multiple frequency bands strongly suggests the left superior cerebellar peduncle may serve as a pivotal node in the neuropathological mechanism of PSCI.

**FIGURE 1:**
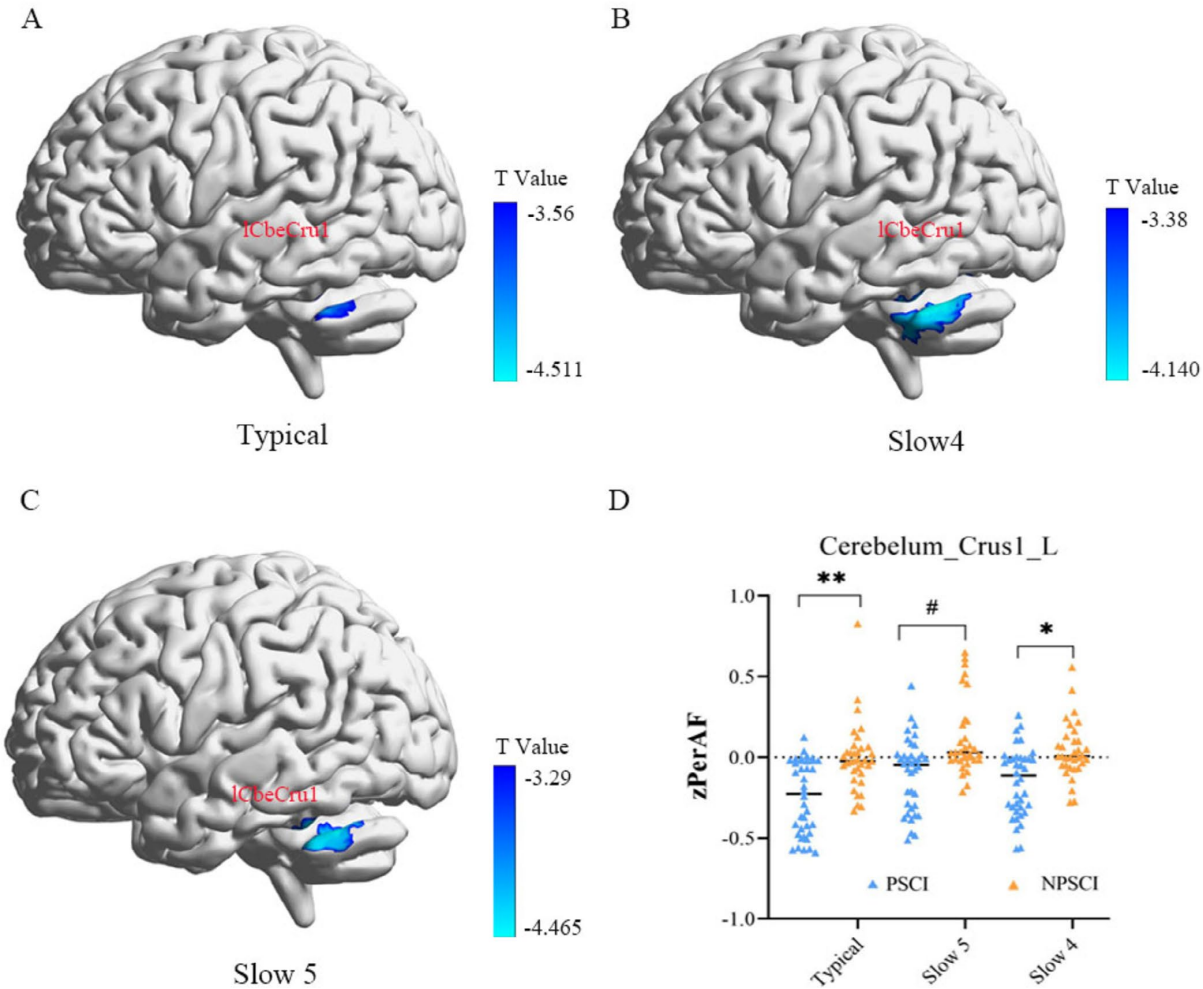
Regional PerAF differences between PSCI and NPSCI groups. Two‐sample *t*‐tests revealed significant between‐group differences in PerAF, particularly in the left cerebellar Crus I (lCbeCru1), highlighted in blue (A–C). As illustrated in (D), normalized PerAF values in the lCbeCru1 were consistently lower in the PSCI group across multiple frequency bands. The color bar represents the *T*‐score scale.

All reported results survived GRF multiple comparison correction (voxel‐level *p* < 0.001, cluster‐level *p* < 0.05). Specific data are provided in Table 2 and Figure 1.

**TABLE 2:** Between‐group differences in PerAF across frequency bands.

Group comparison	Index	Regions (AAL)	MNI coordinate (mm)			Cluster size	Peak *t*‐value
			X	Y	Z		
PSCI < NPSCI	PerAF^a^	lCbeCru1	−39	−54	−27	23	−4.51
	PerAF^b^	lCbeCru1	−39	−54	−27	42	−4.46
	PerAF^c^	lCbeCru1	−39	−54	−27	59	−4.14

*Note:* Regional PerAF values in the left Crus I of the cerebellum (lCbeCru1) were compared between PSCI and NPSCI groups in three frequency bands: (a) conventional band (0.01–0.08 Hz), (b) slow‐4 (0.027–0.073 Hz), and (c) slow‐5 (0.01–0.027 Hz). Significant reductions in PerAF were observed in the PSCI group, highlighting cerebellar Crus I as a key region of oscillatory abnormality in post‐stroke cognitive impairment.

To further investigate the relationship between neural activity in the superior cerebellar peduncle and cognitive function, we constructed 6‐mm spherical regions of interest (ROIs) centered on peak coordinates from the aforementioned analysis. PerAF values within these ROIs were extracted for both the conventional and slow‐4 bands and subjected to partial correlation analysis with MoCA scores. The results demonstrated stable, moderate positive correlations between PerAF in the left superior cerebellar Crus I and MoCA scores across multiple frequency bands in PSCI patients: conventional band (0.01–0.08 Hz): *r* = 0.561, *p* < 0.001 (Figure S1A); slow‐4 band (0.027–0.073 Hz): *r* = 0.506, *p* < 0.001 (Figure S1B). These findings indicate that the strength of low‐frequency oscillatory activity in this region may serve as a potential neuroimaging marker for assessing the severity of cognitive impairment in PSCI.

### 3.3 Dynamic Amplitude of Low‐Frequency Fluctuation in PSCI Patients

**FIGURE 2:**
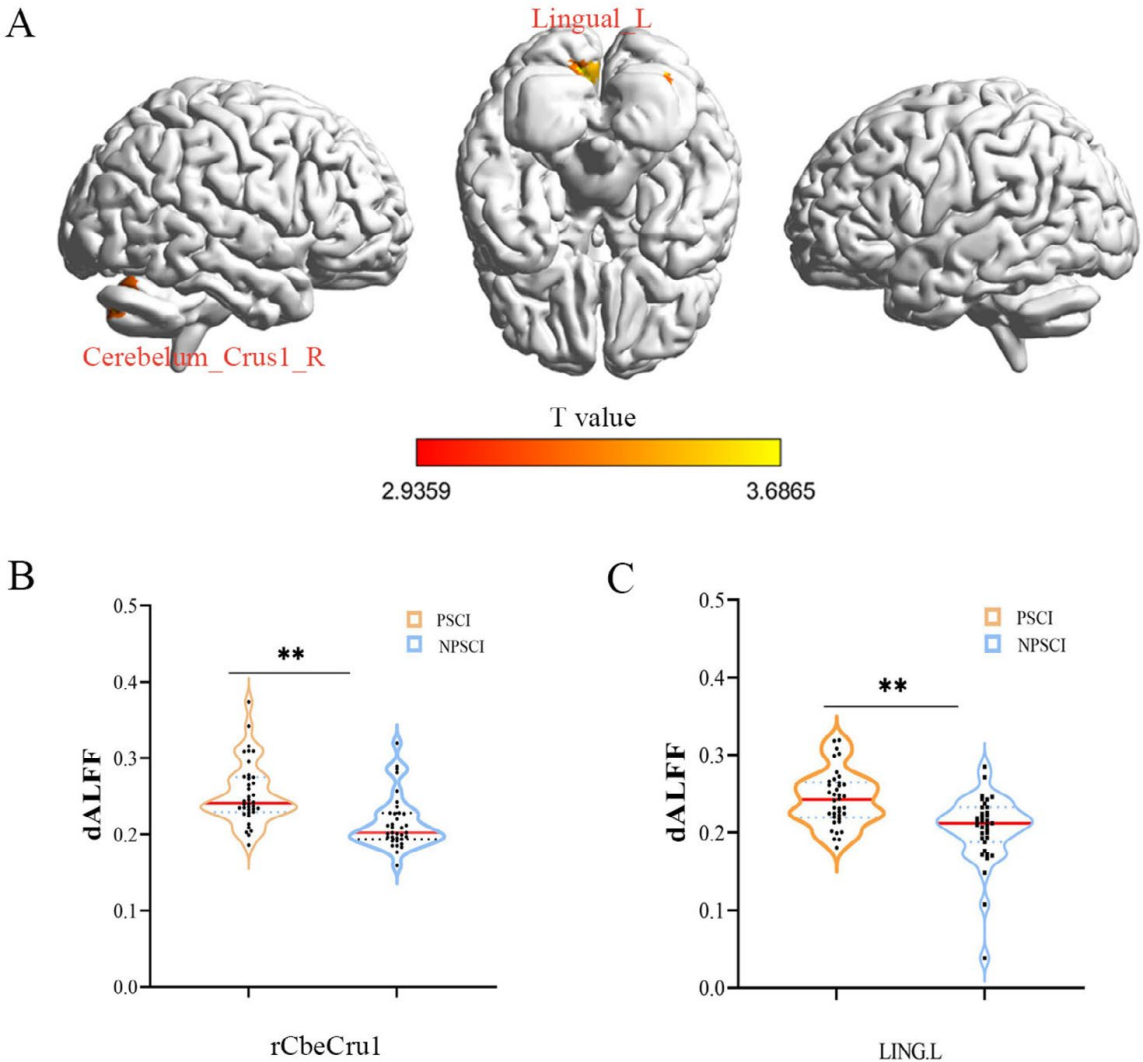
Hyperactive hubs: Cerebellar and lingual gyrus dALFF increases define PSCI. We identified two key hubs of cerebral hyperactivity in PSCI: the right cerebellar Crus I and the left lingual gyrus. The elevated dALFF in these distinct regions (A), quantified in (B) and (C), implicates atypical activity in both cognitive‐associative cerebellar and visual cortical areas as a hallmark of PSCI, pointing to a complex network‐level dysfunction.

To capture the temporal dynamics of spontaneous brain activity, we computed dALFF using a sliding window approach (window length: 50 TRs [100 s], step size: 1 TR [2 s]). After controlling for covariates including age, sex, education level, and mean FD, an independent two‐sample *t*‐test with GRF correction (voxel‐level *p* < 0.005, cluster‐level *p* < 0.05, two‐tailed) revealed significant differences in dALFF between the PSCI and non‐PSCI groups. Brain regions exhibiting aberrant dynamic activity were primarily located in the right cerebellar Crus I (rCbeCru1) and the left lingual gyrus (LING.L). Detailed results are shown in Table S1 and Figure 2.

### 3.4 Dynamic Functional Connectivity in PSCI Patients

**FIGURE 3:**
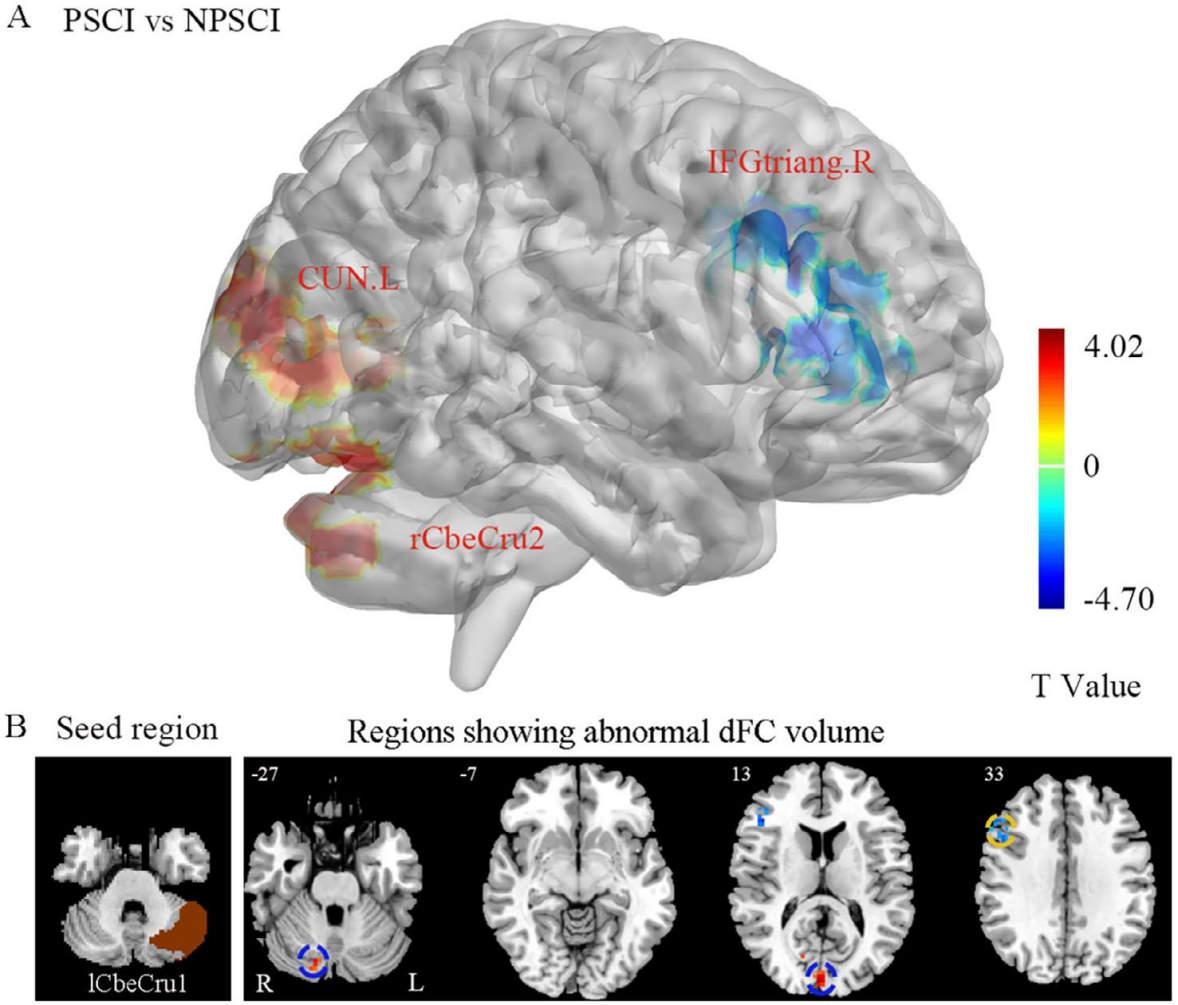
Altered functional connectivity of the left cerebellar Crus I in PSCI. Seed‐based dFC analysis revealed significantly increased connectivity between the left cerebellar Crus I (lCbeCru1) and several brain regions in the PSCI group, including the right cerebellar Crus II (rCbeCru2), right triangular part of the inferior frontal gyrus (IFGtriang.R), and left cuneus (CUN.L) (regions in red). The color bar represents the *T*‐score scale.

**FIGURE 4:**
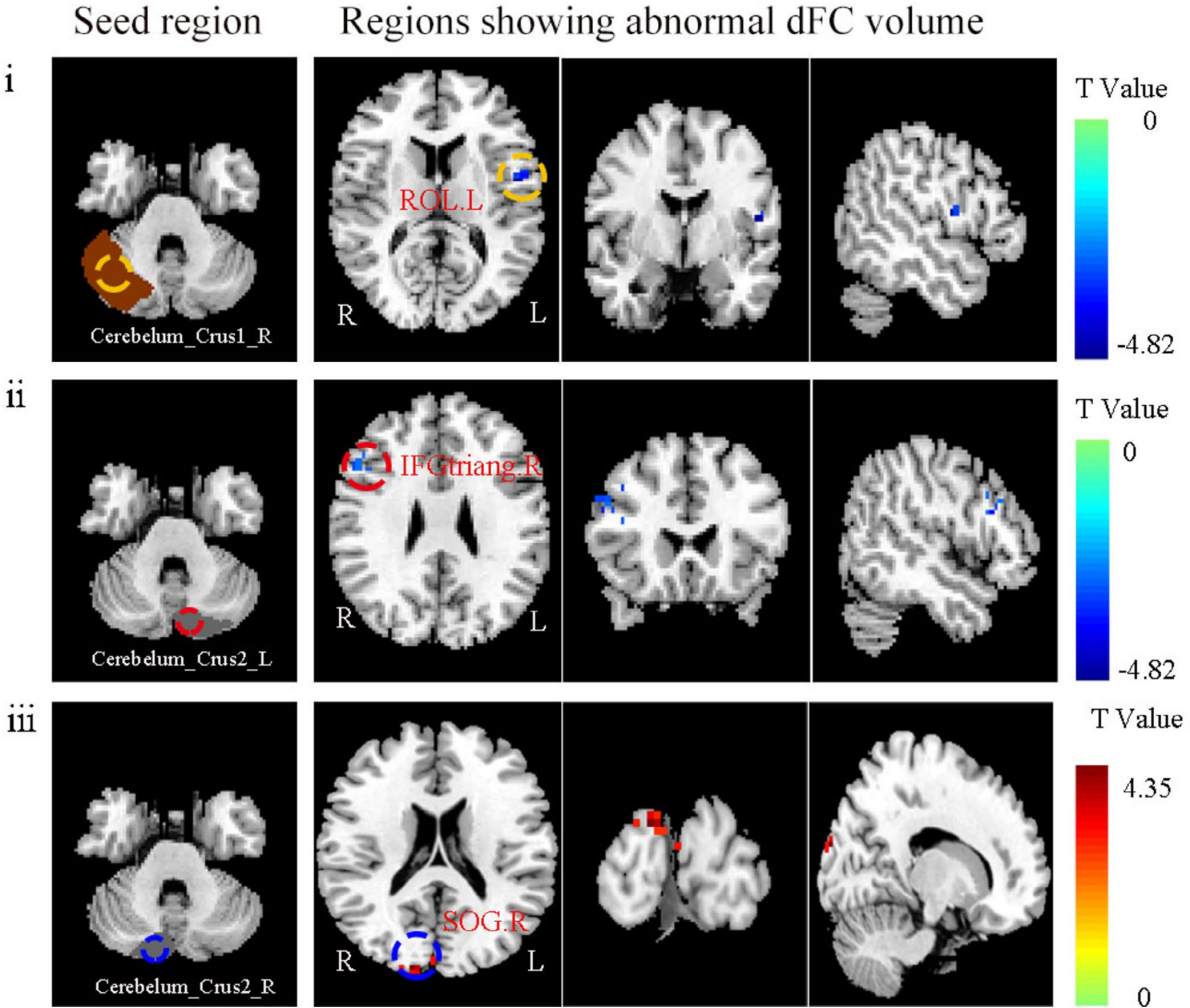
Distinct dFC alterations from cerebellar seeds in PSCI. Seed‐based dFC analyses revealed distinct patterns of cerebellar‐cerebral dysconnectivity in PSCI. (i) Using the right cerebellar Crus I (rCbeCru1) as a seed revealed altered connectivity with the left Rolandic operculum (ROL.L). (ii) Seeding from the left cerebellar Crus II (lCbeCru2) identified changes in connectivity with the right triangular part of the inferior frontal gyrus (IFGtriang.R). (iii) Seeding from the right cerebellar Crus II (rCbeCru2) showed aberrant connectivity with the right superior occipital gyrus (SOG.R). The color bar represents the *T*‐score scale.

To systematically characterize the spatiotemporal dynamics of functional connectivity in PSCI patients, we selected six seed regions closely associated with the cerebellar cognitive network (lCbeCru1, rCbeCru1, lCbeCru2, rCbeCru2, lCbe9, rCbe9) and constructed whole‐brain voxel‐wise dFC maps for each seed. The analyses employed a sliding window method (window: 50 TRs, step: 1 TR) and were adjusted for age, sex, education, and mean FD. Independent two‐sample *t*‐tests with GRF correction (voxel‐level *p* < 0.005, cluster‐level *p* < 0.05, two‐tailed) identified significant between‐group differences in dFC within several cerebro‐cerebellar circuits in PSCI patients compared to the non‐PSCI group. Specific brain regions and statistical details are provided in Table 3, Figures 3 and 4, and Figures S2 and S3.

**TABLE 3:** Aberrant dynamic functional connectivity in PSCI.

Seed	Regions (AAL)	MNI coordinate (mm)			Cluster size	Peak *t*‐value
		X	Y	Z		
lCbeCru1	rCbeCru2	15	−90	−33	69	4.015
	IFGtriang.R	51	24	24	91	−4.704
	CUN.L	0	−93	15	74	3.841
rCbeCru1	ROL.L	−51	−3	12	15	−4.820
lCbeCru2	IFGtriang.R	45	21	21	33	−4.817
rCbeCru2	SOG.R	15	−96	21	28	4.348
lCbe9	STG.L	−66	−12	12	37	−3.827
rCbe9	PCL.L	−6	−36	75	48	−5.585

*Note:* This table catalogues the components of a distributed network showing altered dFC in PSCI patients. The network spans cognitive‐associative cerebellar hubs (Crus I/II, lobule IX) and functionally diverse cerebral areas, implicating disrupted communication across frontal, sensory, visual, and auditory processing circuits.

Abbreviations: AAL, Anatomical Automatic Labeling; CUN.L, left cuneus; dFC, dynamic functional connectivity; IFGtriang.R, right inferior frontal gyrus, triangular part; lCbe9, left cerebellar lobule IX; lCbeCru1, left Crus I; lCbeCru2, left Crus II; MNI, Montreal Neurological Institute; NPSCI, non‐post‐stroke cognitive impairment; PCL.L, left paracentral lobule; PSCI, post‐stroke cognitive impairment; rCbe9, right cerebellar lobule IX; rCbeCru1, right Crus I; rCbeCru2, right Crus II; ROL.L, left rolandic operculum; SOG.R, right superior occipital gyrus; STG.L, left superior temporal gyrus.

Furthermore, based on peak coordinates from regions showing significant group differences in dFC, we defined 6‐mm spherical ROIs and extracted dFC values within these ROIs for both PSCI and non‐PSCI patients. Partial correlation analysis with clinical cognitive scores revealed that for dFC analysis using lCbe9 as a seed, PSCI patients showed significantly reduced functional connectivity in the left superior temporal gyrus (STG.L). This connectivity strength was positively correlated with MoCA scores (*r* = 0.388, *p* = 0.018), suggesting that impaired dynamic functional connectivity between the cerebellum and the temporal lobe may play an important role in the cognitive deficits observed in PSCI (Figure S4).

### 3.5 Computer‐Aided Diagnosis of PSCI Based on Multimodal Neuroimaging Features

**FIGURE 5:**
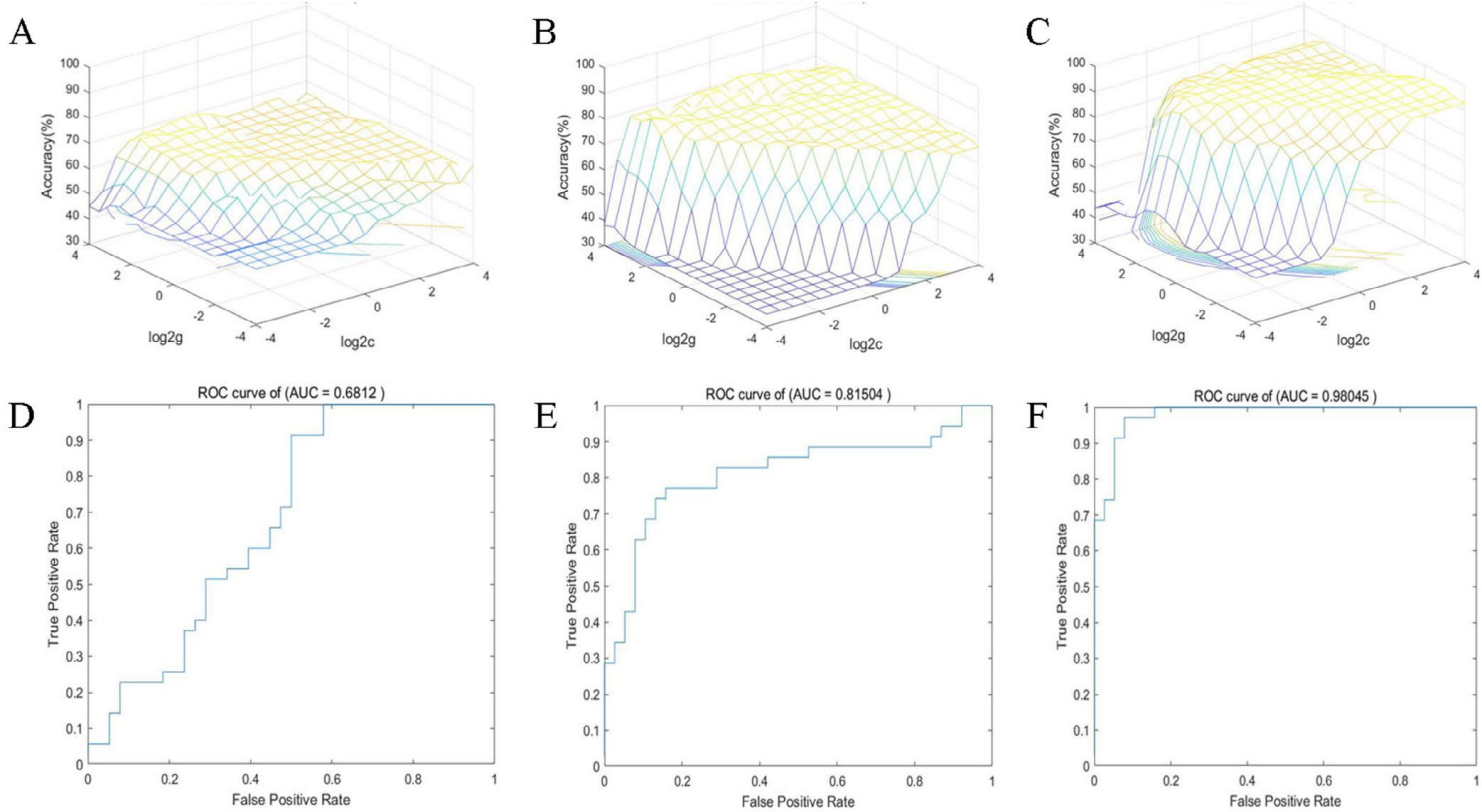
Comparative biomarker performance in SVM classification. SVM models leveraging dynamic functional connectivity (dFC) demonstrated superior classification performance (C, F), outperforming models based on regional indices PerAF (A, D) and dALFF (B, E). For each biomarker, the grid‐search optimized parameters (top) and validation ROC curves (bottom) are shown, underscoring dFC as a highly discriminative feature for identifying disease‐specific neural signatures.

**FIGURE 6:**
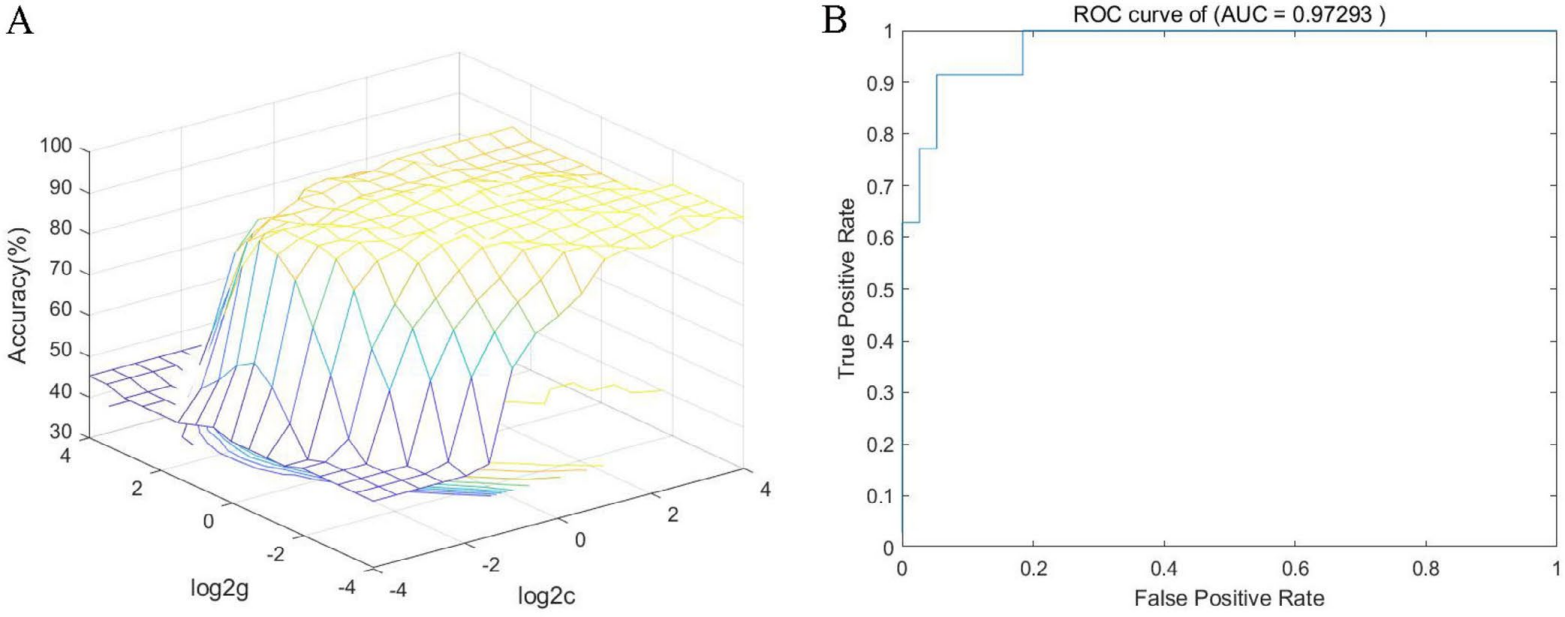
Enhanced diagnostic classification using combined biomarkers. Integration of multimodal neuroimaging metrics (PerAF, dALFF, dFC) yields a powerful classifier for PSCI. The SVM model, optimized via grid search (A), achieves superior discriminatory performance, as evidenced by the ROC curve in (B), outperforming models based on single metrics.

To evaluate the potential clinical utility of the extracted neuroimaging features for identifying PSCI, we constructed an SVM classifier and performed blinded classification of PSCI versus non‐PSCI subjects based on different modal indicators. The following results were obtained:

1. The SVM model based on PerAF values from the left superior cerebellar peduncle achieved an accuracy of 68.49%, sensitivity of 91.43%, specificity of 47.37%, precision of 61.54%, and AUC of 0.68 (Figure 5A,D).
2. Incorporating dALFF features from the right superior cerebellar peduncle and left lingual gyrus significantly enhanced the classification performance, yielding an accuracy of 80.82%, sensitivity of 77.14%, specificity of 84.21%, precision of 81.82%, and AUC of 0.82 (Figure 5B,E).
3. Using dFC features alone achieved excellent classification, with an accuracy of 94.52%, sensitivity of 97.14%, specificity of 92.11%, precision of 91.89%, and AUC of 0.98 (Figure 5C,E).
4. The integrated model combining multimodal features (PerAF + dALFF + dFC) demonstrated stable and superior diagnostic performance, with an accuracy of 93.15%, sensitivity of 91.43%, specificity of 94.72%, precision of 94.12%, and an AUC of 0.97 (Figure 6A,B).

These results are detailed in Table 4 and Figures 5 and 6. Collectively, they indicate that neuroimaging markers based on dynamic functional properties of the cerebellum, particularly when utilizing dFC or a multimodal fusion strategy, exhibit highly promising potential for the computer‐aided diagnosis of PSCI.

**TABLE 4:** SVM classification performance of single and combined metrics.

Variable	PerAF	dALFF	dFC	PerAF+ dALFF+ dFC
Best c	0.25	8	2	0.50
Best g	11.31	0.71	1.41	0.35
Accuracy (%)	68.49	80.82	94.52	93.15
Sensitivity (%)	91.43	77.14	97.14	91.43
Specificity (%)	47.37	84.21	92.11	94.72
Precision (%)	61.54	81.82	91.89	94.12
AUC	0.68	0.82	0.98	0.97

*Note:* The support vector machine (SVM) model was optimized via grid search for the penalty parameter (c) and the radial basis function kernel width (*γ*). Performance was evaluated using standard metrics, including accuracy, sensitivity, specificity, precision, and the area under the curve (AUC). The results demonstrate the diagnostic power of individual and combined neuroimaging metrics in distinguishing PSCI patients.

## 4 Discussion

This study establishes a multidimensional framework that integrates functional segregation metrics (PerAF, dALFF) with a functional integration measure (dFC) to systematically examine cerebral functional reorganization in patients with PSCI following acute BGCI. By concurrently assessing brain network dynamics from both segregation and integration perspectives, this approach offers a more holistic understanding of PSCI pathophysiology. Complementing this, a machine learning paradigm was incorporated to evaluate the diagnostic utility of these neuroimaging features, forming an end‐to‐end analytical pipeline from mechanism exploration to clinical application.

Guided by this framework, our results revealed a distinctive pattern of functional disturbances in PSCI. We observed significantly reduced PerAF in the lCbeCru1, indicating compromised neural consistency, co‐occurring with markedly elevated dALFF in the rCbeCru1and left lingual gyrus (LING.L), suggestive of localized compensatory or pathological hyperactivity. Extending beyond these regional anomalies, dFC analysis revealed a broader network‐level imbalance, featuring strengthened connectivity in cerebellar‐visual pathways (e.g., between rCbeCru2 and CUN.L) alongside widespread weakening within key cognitive cortical networks, including the inferior frontal and superior temporal gyri.

To assess the clinical applicability of these neuroimaging patterns, we employed machine learning for diagnostic validation. The SVM model utilizing dFC features demonstrated outstanding classification performance (AUC = 0.98, accuracy = 94.52%), substantially surpassing models based on single segregation metrics. Notably, integrating multimodal features further enhanced precision to 94.12%, highlighting the superior discriminative capability of a combined metric approach and reinforcing the potential of dFC as a highly sensitive biomarker for PSCI.

Collectively, these findings advance a refined and coherent model of PSCI as a network‐level disorder, characterized by a dual phenomenon of cerebellar‐visual compensation and cortical network disintegration. The identified neuroimaging signatures are a promising set of biomarkers for early PSCI detection. Furthermore, the robust performance of our machine learning framework outlines a practical pathway toward precision medicine in cerebrovascular cognitive management, establishing a foundation for developing targeted intervention strategies.

### 4.1 Frequency‐Specific PerAF Differences Between Groups

PerAF is an emerging fMRI analytical metric that reliably reflects the amplitude of BOLD signal fluctuations over time, thereby characterizing the stability of neural activity in local brain regions. This study found that PerAF in lCbeCru1 was consistently lower across three frequency bands—the conventional band, slow‐4, and slow‐5—in the PSCI group compared to the non‐PSCI group. Furthermore, its value showed a moderate positive correlation with MoCA scores. Crus I and Crus II are key nodes within the cerebro‐cerebellar “cognitive loop” and play a central role in cognitive processes such as executive function, working memory, and strategic planning [12, 37, 38]. Recent neuromodulation studies have also indicated that theta‐burst stimulation (TBS) targeting the right Crus I can improve language function in patients with post‐stroke aphasia [39]. Our study provides novel evidence from the perspective of local neural activity stability for the role of the cerebellum in PSCI pathogenesis, offering a theoretical basis for future cerebellar‐targeted neuromodulation therapies for PSCI.

### 4.2 dALFF Differences in PSCI Patients

dALFF captures the dynamic temporal variations of spontaneous brain neural activity, serving as an important complement to the traditional ALFF metric [40]. The current study found that PSCI patients exhibited significantly increased dALFF in the right cerebellar Crus I and the left lingual gyrus. Previous studies have confirmed that Crus I/II is anatomically and functionally connected to the prefrontal cortex, participating in cognitive and emotional regulation [41]. Disruption of functional connectivity within the frontal‐cerebellar circuit is closely associated with clinical manifestations in various cognitive disorders [42]. Our results further suggest that focal structural damage caused by BGCI may indirectly lead to cognitive dysfunction by affecting the dynamic activity of cognitive networks. Additionally, increased dALFF in the left lingual gyrus might reflect compensatory functional reorganization of the visual processing center post‐stroke. As a key region for the integration of visual information, the lingual gyrus plays vital roles in visual memory, creative thinking, and language processing [43, 44]. Its heightened activity could represent an adaptive remodeling mechanism aimed at compensating for insufficient cognitive resources [45]. Therefore, it is reasonable to speculate that if a stroke involves or affects the neural network associated with the left lingual gyrus, this may lead to impairment in related cognitive domains such as visuospatial memory and semantic processing. Conversely, the preservation of this region or successful functional reorganization may support cognitive recovery [46]. Further research is needed to directly validate the value of the left lingual gyrus as a prognostic biomarker or as a target for neuromodulation therapy in populations with PSCI [47].

### 4.3 Alterations in dFC in PSCI Patients

By employing dynamic functional connectivity analysis (window length 50 TRs, step size 1 TR), this study found that intra‐hemispheric dFC alterations in PSCI patients primarily involved connections between the left cerebellar Crus I and left Crus II, the left cerebellar Crus I and left cuneus, and the left lobule IX and right superior temporal gyrus. Crus I/II are core components of the posterior cerebellar lobe and exhibit extensive connections with the DMN [11, 48]. The observed increased dFC variability between Crus I and visual‐related regions like the cuneus suggests enhanced dynamic collaboration between the cerebellar and visual networks post‐stroke, potentially constituting a compensatory mechanism for integrating cognitive and visual information. Concurrently, the decreased dFC in the frontal, temporal, and cuneus regions in PSCI patients reflects weakened interaction between the DMN and the frontoparietal network, further supporting the crucial role of network‐level dysregulation in PSCI pathogenesis [49]. The posterior cerebellar lobe, including Crus I/II and lobule IX, is a key area of the “cognitive cerebellum,” and the reorganization of its dynamic connectivity with large‐scale whole‐brain networks may offer a novel perspective for understanding the neural mechanisms of PSCI [50]. Thus, our study advances the prevailing model of PSCI from a primarily cortical‐DMN disorder to a more integrated “cerebro‐cerebellar network dysfunction” model. This cerebellar perspective provides a novel mechanistic link, suggesting that PSCI may arise not only from direct cortical network damage but also from disrupted subcortical–cortical communication and compensatory cross‐network recruitment.

### 4.4 Computer‐Aided Diagnostic Classification Based on SVM Model

Results from the SVM model revealed that the best performance was obtained with the classifier based on dFC features (AUC = 0.98). Multimodal feature fusion further improved the classification precision (94.12%). While the multi‐feature model showed a marginal dip in overall accuracy, its superior precision and specificity are clinically paramount. This performance profile significantly reduces false positives, thereby minimizing misdiagnosis and preventing unnecessary interventions. Consequently, it offers a more reliable tool for clinicians to confidently identify high‐probability PSCI cases and make targeted referral or treatment decisions. Possible explanations for the superior performance of the single‐feature dFC model include: (1) inherent redundancy among features—since PerAF, dALFF, and dFC all originate from the same BOLD signal, dFC may already reflect much of the information captured by the other two; (2) limitations of the linear SVM, which may not adequately model nonlinear complementarity between multimodal features; and (3) the effect of restrictive feature selection, which could have excluded subtle yet contributory signals from PerAF and dALFF. Nevertheless, the combined‐feature model achieved the highest precision (94.12%) and specificity (94.72%), suggesting these features still hold complementary clinical value not fully utilized under the current linear fusion framework. This underscores the significant discriminatory value of dynamic connectivity patterns between cerebellar cognitive seeds and the whole brain in distinguishing PSCI from non‐PSCI, particularly features involving the right Crus II, left cuneus, and left superior temporal gyrus. This study established a rapid, automated auxiliary diagnostic framework that holds promise for improving the early identification efficiency of PSCI in future clinical practice.

We acknowledge that precise infarct sublocation, volume, and individual medication details can potentially influence functional networks. Our study aimed to mitigate these confounders through stringent radiological selection and standardized clinical management. The findings should therefore be interpreted as reflecting PSCI‐associated network dysfunction within the context of acute BGCI, while recognizing the possible contributory role of subtler, unmeasured clinical‐anatomical variables.

## 5 Limitations and Future Directions

This study has several limitations. First, the relatively small sample size restricts our ability to perform in‐depth subgroup analyses, such as comparisons across patients with different levels of cognitive impairment severity. Relatedly, we did not stratify PSCI patients based on severity (e.g., mild cognitive impairment versus major neurocognitive disorder). This lack of stratification may limit a nuanced understanding of how the identified neuroimaging biomarkers relate to disease severity and could also affect the generalizability of our findings to PSCI populations with varying degrees of cognitive deficit. Future studies should incorporate more refined clinical stratification to assess the discriminative utility of these biomarkers across distinct PSCI severity subgroups. Second, the cross‐sectional study design precludes causal inferences regarding the relationship between the neuroimaging metrics and the progression or prognosis of PSCI. Third, although the machine learning model was internally validated using cross‐validation, it has not yet been tested in an independent external cohort. Thus, its generalizability remains to be confirmed. Future work should employ larger, multi‐center prospective cohorts with longitudinal follow‐up to verify the predictive value of these imaging biomarkers and to rigorously evaluate externally validated SVM models, thereby facilitating their translation into clinical diagnostic tools. Finally, this study did not perform detailed infarct subnuclei mapping/volumetry or include specific medication regimens as covariates in the analysis. Although both groups received similar standardized care, potential individual variations in pharmacological effects cannot be entirely ruled out. Future studies incorporating these refined measures would help to further elucidate the determinants of post‐stroke network alterations.

## 6 Conclusions and Implications

By integrating multimodal functional imaging metrics, this study systematically revealed significant abnormalities in both functional segregation and integration within multiple brain regions, including the cerebellum, DMN, and visual cortex, in patients with PSCI following acute BGCI. We also preliminarily constructed an SVM‐based auxiliary diagnostic model, providing new methods and perspectives for researching the neural mechanisms and early identification of PSCI. The findings indicate that alterations in metrics such as PerAF, dALFF, and dFC in specific brain regions can serve as potential imaging markers for differentiating between PSCI and non‐PSCI patients. Furthermore, functional reorganization of the cerebellum and its associated networks is likely to play a critical role in cognitive compensation after stroke.

## Author Contributions

Shijian Chen, Jian Zhang, and Liya Pan contributed equally to this work. The article is mainly conceived and written by Shijian Chen and Jian Zhang. Baohui Weng and Yijie Mo performed the literature search. Xuemei Quan, Gengyu Cen, and Xize Jia designed the figure and table. Liya Pan and Zhijian Liang contributed to manuscript revisions. Yayuan Liu and Zhijian Liang designed and supervised this work. All authors have read and approved the final submission.

## Funding

This study was supported by the Guangxi Natural Science Foundation (2025GXNSFAA069038) and the Liuzhou Science and Technology Bureau (2024YB0103A014). Additional support was provided by grants from the National Natural Science Foundation of China (82260243, 82560240).

## Disclosure

Guarantor: The scientific guarantor of this publication is Zhijian Liang.

## Ethics Statement

Studies involving human patients were reviewed and approved by the Guangxi Medical University Review Board (Reference Number: 2021 KY‐E‐184).

## Consent

Informed consent was obtained from all subjects involved in the study. This project fully considered and protected the rights and interests of the study objects. It meets the criteria of the Ethical Review Committee. The Medical Ethics Committee of First Affiliated Hospital of Guangxi Medical University has approved the protocol.

## Conflicts of Interest

The authors declare no conflicts of interest.

## Supporting information


**Table S1:** Between‐group differences in dALFF.
**Figure S1:** Cerebellar PerAF correlates with cognitive performance in PSCI. PerAF in the left Crus I of the cerebellum (lCbeCru1) shows a significant positive correlation with MoCA scores in PSCI patients. This relationship is observed both in the conventional frequency band (A) and the slow‐4 sub‐band (B), suggesting that reduced cerebellar fluctuation amplitude is linked to poorer cognitive function. lCbeCru1, left Crus I of the cerebellum; MoCA, Montreal Cognitive Assessment; PerAF, percent amplitude of fluctuation; PSCI, post‐stroke cognitive impairment.
**Figure S2:** Altered dFC from cerebellar lobule IX in PSCI. Seed‐based dFC analysis revealed significant between‐group differences. (i) Using the left cerebellar lobule IX (lCbe9) as a seed identified altered connectivity with the left superior temporal gyrus (STG.L). (ii) Seeding from the right cerebellar lobule IX (rCbe9) identified altered connectivity with the left paracentral lobule (PCL.L). The color bar represents the *T*‐score scale.
**Figure S3:** Widespread cerebellar‐cortical dysconnectivity in PSCI. Our findings delineate a distributed network of dysconnectivity in PSCI. Panel (a) provides an overview of the aberrant cerebellar‐cerebral circuits identified. Quantitative comparisons in (b) and (c) confirm that PSCI patients exhibit significantly altered dFC across multiple systems, involving frontal executive (IFGtriang.R), somatosensory (ROL.L, PCL.L), and visual processing (CUN.L, SOG.R) regions. This pattern implicates the cerebellum as a key node whose disrupted communication with diverse cortical networks underpins cognitive deficits. *Significance: *p < 0.05, *p < 0.01 vs. NPSCI; #*p* < 0.05, ##*p* < 0.01 for other comparisons.
**Figure S4:** Correlation between left superior temporal gyrus connectivity and cognitive function. Scatter plot illustrating the significant correlation between dFC values of the left superior temporal gyrus (STG.L) and MoCA scores in the PSCI cohort. This association suggests a link between auditory and language processing network integrity and global cognitive performance post‐stroke.

## Data Availability

The data that supports the findings of this study are available in the Supporting Information of this article.
