# Supplementary material for: Decoding Post‐Stroke Cognitive Impairment After Acute Basal Ganglia Infarction: The Synergistic Role of Functional Segregation and Integration in an SVM fMRI Framework
Source: CNS Neurosci Ther. 2026 Apr 10;32(4):e70871. doi: 10.1002/cns.70871 (PMC13068037; doi:10.1002/cns.70871)
Supplement: Supplementary file 1 — Table S1: Between‐group differences in dALFF. Figure S1: Cerebellar PerAF correlates with cognitive performance in PSCI. PerAF in the left Crus I of the cerebellum (lCbeCru1) shows a significant positive correlation with MoCA scores in PSCI patients. This relationship is observed both in the conventional frequency band (A) and the slow‐4 sub‐band (B), suggesting that reduced cerebellar fluctuation amplitude is linked to poorer cognitive function. lCbeCru1, left Crus I of the cerebellum; MoCA, Montreal Cognitive Assessment; PerAF, percent amplitude of fluctuation; PSCI, post‐stroke cognitive impairment. Figure S2: Altered dFC from cerebellar lobule IX in PSCI. Seed‐based dFC analysis revealed significant between‐group differences. (i) Using the left cerebellar lobule IX (lCbe9) as a seed identified altered connectivity with the left superior temporal gyrus (STG.L). (ii) Seeding from the right cerebellar lobule IX (rCbe9) identified altered connectivity with the left paracentral lobule (PCL.L). The color bar represents the T‐score scale. Figure S3: Widespread cerebellar‐cortical dysconnectivity in PSCI. Our findings delineate a distributed network of dysconnectivity in PSCI. Panel (a) provides an overview of the aberrant cerebellar‐cerebral circuits identified. Quantitative comparisons in (b) and (c) confirm that PSCI patients exhibit significantly altered dFC across multiple systems, involving frontal executive (IFGtriang.R), somatosensory (ROL.L, PCL.L), and visual processing (CUN.L, SOG.R) regions. This pattern implicates the cerebellum as a key node whose disrupted communication with diverse cortical networks underpins cognitive deficits. *Significance: *p < 0.05, *p < 0.01 vs. NPSCI; #p < 0.05, ##p < 0.01 for other comparisons. Figure S4: Correlation between left superior temporal gyrus connectivity and cognitive function. Scatter plot illustrating the significant correlation between dFC values of the left superior temporal gyrus (STG.L) and MoCA scores in the PS [file CNS-32-e70871-s001.zip › cns70871-sup-0002-FigureS1-S4@Figure captions S.docx]

**Fig. S1 | Cerebellar PerAF correlates with cognitive performance in PSCI**

**Note:**PerAF in the left Crus I of the cerebellum (lCbeCru1) shows a significant positive correlation with MoCA scores in PSCI patients. This relationship is observed both in the conventional frequency band (A) and the slow-4 sub-band (B), suggesting that reduced cerebellar fluctuation amplitude is linked to poorer cognitive function. PSCI, post-stroke cognitive impairment; PerAF, percent amplitude of fluctuation; lCbeCru1, left Crus I of the cerebellum; MoCA, Montreal Cognitive Assessment.

**Fig. S2 | Altered dFC from cerebellar lobule IX in PSCI**

**Note:** Seed-based dFC analysis revealed significant between-group differences. (i) Using the left cerebellar lobule IX (lCbe9) as a seed identified altered connectivity with the left superior temporal gyrus (STG.L). (ii) Seeding from the right cerebellar lobule IX (rCbe9) identified altered connectivity with the left paracentral lobule (PCL.L). The color bar represents the T-score scale.

**Fig. S3 | Widespread cerebellar-cortical dysconnectivity in PSCI**

**Note:** Our findings delineate a distributed network of dysconnectivity in PSCI. Panel (a) provides an overview of the aberrant cerebellar-cerebral circuits identified. Quantitative comparisons in (b) and (c) confirm that PSCI patients exhibit significantly altered dFC across multiple systems, involving frontal executive (IFGtriang.R), somatosensory (ROL.L, PCL.L), and visual processing (CUN.L, SOG.R) regions. This pattern implicates the cerebellum as a key node whose disrupted communication with diverse cortical networks underpins cognitive deficits.

*Significance: **p* < 0.05, **p* < 0.01 vs. NPSCI; #*p* < 0.05, ##*p* < 0.01 for other comparisons.

**Fig. S4 | Correlation between left superior temporal gyrus connectivity and cognitive function**

**Note:** Scatter plot illustrating the significant correlation between dFC values of the left superior temporal gyrus (STG.L) and MoCA scores in the PSCI cohort. This association suggests a link between auditory and language processing network integrity and global cognitive performance post-stroke.
